# Studies on Agrochemical Controlled Release Behavior of Copolymer Hydrogel with PVA Blends of Natural Polymers and Their Water-Retention Capabilities in Agricultural Soil

**DOI:** 10.3390/polym15173545

**Published:** 2023-08-25

**Authors:** Fawzi Habeeb Jabrail, Maysam Salih Mutlaq, Roua’a Kassim Al-Ojar

**Affiliations:** 1 Polymer Research Laboratory, Department of Chemistry, College of Science, University of Mosul, Mosul 41002, Iraq; mutlaqmaysam@gmail.com; 2 College of Pharmacy, Nineveh University, Mosul 41002, Iraq; roua_al_awjar@yahoo.com

**Keywords:** controlled agrochemical release, urea fertilizer, poly(vinyl alcohol), chitosan, gum Arabic, SAP hydrogel, water retention

## Abstract

Agricultural technical development relies exclusively on the effective delivery of agrochemicals and water to plants and on reducing the harmful effects of agrochemicals on useful organisms in the soil. In this study, super-absorbent hydrogels were prepared in the form of microspheres using gum Arabic (GA), which was copolymerized once with chitosan (CS) and once with poly (vinyl alcohol) (PVA). To impart mechanical strength to the hydrogel microspheres, a covalent cross-linker (N,N′-methylenebisacrylamide (MBA)) was used for the PVA/GA hydrogel, and an ionic cross-linker (sodium hexametaphosphate (SHMP)) was used for the CS/GA hydrogel. The prepared PVA/GA-CH and CS/GA-PH hydrogel microspheres showed different degrees of swelling (DSs) in the following solution media: deionized water (DW), river water (RW), and buffered solutions (pH 4; pH 9). The PVA/GA-CH hydrogel microspheres showed a maximum DS of 84 g/g in the RW, while the CS/GA-PH hydrogel microspheres showed a maximum DS of 63 g/g in the buffered solution at a pH 9. The water-retention capabilities of the hydrogels were studied using a mixture of 0.5% (*w*/*w*) hydrogel microspheres in agricultural soil; the composite showed an additional 20 days of water retention in comparison with a control sample consisting of soil alone. The hydrogels were loaded with urea, which is an important fertilizer in the field of agriculture. The PVA/GA-CH hydrogel microspheres showed a maximum loading percentage (L_max_%) of 89% (*w*/*w*), while the CS/GA-PH hydrogel microspheres showed an L_max_% = 79.75% (*w*/*w*) for urea. The urea-release behaviors of the hydrogel microspheres were studied under different release media and temperature conditions. In practice, the PVA/GA-CH hydrogel microspheres showed a better release profile in the RW at 10 °C, while the CS/GA-PH hydrogel microspheres showed a more controlled release in media at a pH 9 and at 30 °C. The urea-loaded microspheres, aside from those following the release, were characterized via FTIR and SEM. In contrast, virgin microspheres were characterized using XRD,^1^H NMR, (TGA and DSC), and the maximum degree of swelling, in addition to being subjected to SEM and FTIR analyses.

## 1. Introduction

Super-absorbent polymers (SAPs) are macromolecules with long chains that have soft network structures and a high ability to absorb a significant amount of water due to their three-dimensional structures [1].

Recently, many studies have utilized hydrogels, which have received a significant amount of attention in agricultural applications, as water-management polymers that reduce the consumption of irrigation water and control the release of different fertilizers into the soil [2,3]. Hydrogels have the ability to retain water in high quantities; therefore, hydrogels can be used in agricultural irrigation, especially in agricultural lands in arid and semi-arid regions [4]. Improvements in soil and its fertility are achieved by increasing the soil water content and improving the uptake of nutrients by plants [5]. Generally, under drought conditions, hydrogels can absorb large quantities of water via irrigation or during rainfall and act as water reservoirs [6,7]. Mixing hydrogels with the soil in an agricultural field could increase the moisture content in the soil, which is important for plants. Similarly, the hydrogels could release fertilizers into the soil over a longer period of time [8]. The high swelling rates and amounts of water absorbed by agricultural hydrogels with high-level mechanical properties are important characteristics that depend on the nature of the hydrogels’ monomers and the polymerization processes used [9].

Hydrogels made from synthetic polymers possess many tunable properties, but they sometimes have limitations; therefore, they are modified chemically with natural polymers in order to obtain precisely controlled micro-environments and bioactive features that originate from natural polymers [10]. Therefore, PVA the synthetic cationic polymer of basic nature with toughness and wear resistance properties was suggested as good candidate for preparing hydrogel microspheres for agricultural uses especially its highly heat and light stability [11]. Moreover, bioactive polysaccharides were copolymerized with PVA to improve the physicochemical and mechanical properties of the final copolymer. Gum Arabic, an anionic polysaccharide with a gelling character, is stable and hard in form. This polysaccharide has moisture-retention and water-binding functionalities that can assist microspheres in retaining water for a long time [12]. In addition, chitosan, a cationic polysaccharide which is biodegradable and has the ability to withstand an applied load without failure, which is a good property for hydrogel microspheres, can be mixed with agricultural soil in fields. Moreover, chitosan can form strong hydrogen bonding between the chains due to its liner chain conformation, therefore exhibits excellent fiber-forming ability [13].

In this study, different hydrogels were prepared via the copolymerization of natural polymers with a synthetic polymer. The goals of this study were to prepare hydrogel microspheres for the sustained release of agrochemicals into agricultural soil and to confirm their suitable loading and releasing conditions. The other important goal was to examine the tendency of the hydrogel microspheres to absorb water and to study their water retention over a long period of time before it is released into soil for the irrigation of plants. The gum Arabic was copolymerized with chitosan and poly (vinyl alcohol), and the prepared hydrogels were characterized for their structural, thermal, crystalline, and morphological properties; their characterization is considered very important for the safety of the hydrogel particles, especially as they will be used in agricultural lands. Therefore, ^1^H NMR, FTIR (TGA and DSC), XRD, and SEM analyses were used. The degrees of swelling of the hydrogel microspheres were studied, and they were then loaded with urea and finally allowed to release the urea in different media solutions and at different temperatures.

## 2. Experimental

### 2.1. Materials and Chemicals

The poly(vinyl alcohol) (PVA) was obtained from Fisher Scientific, Waltham, MA, USA. Highly viscose chitosan (CS), and gum Arabic (GA) were obtained from Sigma-Aldrich (Saint Louis, MO, USA). The chitosan (70% DDA) was dissolved in 2% (*w*/*w*) acetic acid for purification and then filtered under pressure to remove undissolved particles of chitosan. The clear filtrate was subsequently precipitated using a 1M NaOH solution and filtered and dried at 25 °C under a vacuum. The compounds N,N′-methylenebisacrylamide (MBA), sodium hexametaphosphate (SHMP), ammonium persulphate (APS), and the urea fertilizer model were obtained from BDH, Brighouse, UK. Phosphate buffered solution pH 4 and boric buffered solution pH 9 and other chemicals were of analytical grade reagents and received from Fluka, Geneva, Switzerland. River water (RW) of a hardness of 250 ppm was collected from the Tigris river in Mosul.

### 2.2. Preparation of (CS-co-GA) and (PVA-co-GA) Copolymers

The chitosan solution was prepared from 1.0 g of CS in 100 mL of 2% (*v*/*v*) acetic acid in distilled water. The gum Arabic solution was prepared from 1.0 g of GA in 100 mL of distilled water. A volume of 10.0 mL of GA solution, 1.0% (*w*/*v*), was heated in a 250 mL three-necked round-bottom flask at 65 °C, using a mantle with a magnetic stirrer. The flask was connected to a nitrogen line passed through an alkaline pyrogallol solution that was free of oxygen. A volume of 5.0 mL of 10.0% (*w*/*v*) APS initiator solution was added, followed by 10.0 mL of a 1.0% (*w*/*v*) CS solution that was added stepwise using a fine-needle syringe, and the temperature of the mixed solution was kept at 65 °C with continuous, quiet stirring. Then, 5.0 ml of 2% (*w*/*v*) of an MBA covalent cross-linker was added with continuous stirring. Finally, 5.0 min later, accompanied by stirring for one hour without heating, the formed CS/GA-CH hydrogel microspheres were filtered, washed many times with distilled water, and finally dried in a vacuum oven at 30 °C.

Whereas, the (PVA-co-GA) hydrogel microspheres were prepared using previous stock solution of 1.0% (*w*/*v*) GA solution. Where, 10.0 mL of 1.0% (*w*/*v*) was heated in 250 mL three-necked round bottom flask at 65 °C using mantle with magnetic stirrer. The flask was connected to a nitrogen line passed through an alkaline pyrogallol solution that was free of oxygen. A volume of 5.0 mL of 10.0% (*w*/*v*) APS initiator solution was added. Then, 20.0 mL of the 1.0% (*w*/*v*) PVA solution was added stepwise, using a fine-needle syringe, and the temperature of the mixed solution was kept at 65 °C with continuous, quiet stirring. A volume of 5.0 mL of 6% (*w*/*v*) SHMP, the ionic cross-linker, was also added stepwise and maintained at 65 °C with stirring. Finally, the formed PVA/GA-PH hydrogel microspheres were kept in a water bath at 80 °C for 24 h before filtration.

### 2.3. Degree of Swelling

A mass of 100 mg of the dry hydrogel microspheres was immersed in 20 mL of one of the following solutions (distilled water (DW), river water (RW), buffered solution (pH 4; pH 9)). The hydrogel was left in the swelling solution for 24 h in order to reach its maximum degree of swelling. The swelling solutions were kept at temperatures of 10 °C, 25 °C, and 50 °C for comparison. After 6 h, the hydrogel microspheres were removed, filtered with a 100-mesh sieve, and left for 10 min inside the sieve for the solution to drain. The swollen hydrogel microspheres were weighted and then returned to the solution. This process was repeated four times after 6 h, and the degree of swelling was calculated using the following equation [14]:

Degree of swelling DS (g/g) = W_t_ − W_0_/W_0_ (1)

where W_t_ is the weight of the wet sample at a time (t), and W_0_ is the weight of the dry sample.

### 2.4. Water-Retention Percentage (WR%) of Soil-Hydrogel Mixture

A mass of 200 g of a dry agricultural soil sample was mixed with 0.1 g of the prepared hydrogel microspheres in a ventilated paper cup. Thereafter, 100 mL of river water (RW), representing the minimum rain fall level recorded in the most arid and semi-arid regions [15], was added slowly to the mixture. A control sample was prepared in a separate cup using the same amount of dry soil and same procedure except no hydrogel microspheres were added. The examined cups were maintained in the same place and under the same conditions, which were a temperature of 20 °C and 25% humidity. The samples were weighted once daily, and the WR% values were calculated as follows [16]:

Water retention (WR) (%) = W_t_/W_0_ × 100 (2)

where W_t_ represents the sample weight after the defined time, and W_0_ is the initial weight.

### 2.5. Loading of Urea Fertilizer on Hydrogel Microspheres

The concentrations of the urea loaded on the hydrogel microspheres were measured using a UV-visible JASCOV-630 spectrophotometer, Tokyo, Japan. The calibration curve was determined from different known concentrations of urea, and the microspheres’ absorbances were measured at λ_max_ 278 nm. The calibration curve was then used to calculate the unknown concentrations of the urea loaded in the hydrogel microspheres. Moreover, 100 mg of the hydrogel microspheres was kept in a 0.1% (*w*/*v*) solution of 100 mg of urea in 100 mL of distilled water. The loading temperature was fixed at 25 °C, room temperature. The loading time was varied between 6, 12, and 24 h in order to record the maximum loading percentage. The following equations were used for calculating the maximum loading percentage (L_max_%) and efficient loading percentage (EL%) [17]:

(3) $$L_{\max}\%=\;\frac{\mathrm{Amount}\;\mathrm{of}\;\mathrm{urea}\;\mathrm{loaded}\;\mathrm{on}\;\mathrm{hydrogel}}{\mathrm{Amount}\;\mathrm{of}\;\mathrm{microspheres}\;\mathrm{taken}\;\mathrm{for}\;\mathrm{loading}}\times 100$$

(4) $$\mathrm{EL}\%=\frac{\;\mathrm{Amount}\;\mathrm{of}\;\mathrm{urea}\;\mathrm{loaded}\;\mathrm{on}\;\mathrm{hydrogel}}{\mathrm{Amount}\;\mathrm{of}\;\mathrm{urea}\;\mathrm{taken}\;\mathrm{for}\;\mathrm{loading}}\times 100$$

### 2.6. Studying the Controlled Release of Urea from Hydrogel Microspheres

Loaded hydrogel microspheres 100 mg was allowed to release in 20 mL solution of DW, RW and buffered solution pH 9. For comparison, the release media were examined at temperatures of 10, 25, and 50 °C. The absorbance of the released urea from the hydrogel microspheres in different release media solutions were measured by UV-Visible spectrophotometer at λ_max_ 278 nm and for a fixed time interval of 3 h by taking 3 mL of solution from release media and replaced with 3 mL of the native solvent. The calibration curve was used to calculate the different unknown release concentrations of urea from their absorption values. The release process was continued until no urea was released. The release of urea was given as the controlled release percentage (CRmax%) using Equation (5) [18]:

(5) $$\mathrm{Controlled}\;\mathrm{release}\;\left(\mathrm{CRmax}\right)\%=\sum {\left(\frac{W_{t}}{W_{o}}\times 100\right)}_{\mathrm{constant}}\;$$

and as the burst release percentage (BRmax%) using Equation (6);

(6) $$\mathrm{Burst}\;\mathrm{release}\;\left(\mathrm{BRmax}\right)\%=\sum {\left(\frac{W_{t}}{W_{o}}\times 100\right)}_{\mathrm{variable}}$$

where $[\frac{\mathrm{Wt}}{W_{o}}$ $\times 100{]}_{t}$ is variable amount for burst release while constant for controlled release.

Using Equation (7), the overall release was calculated as the cumulative release percentage (Rcum%) for a fixed time interval of 3 h:

(7) $$\mathrm{Cumulative}\;\mathrm{release}\;\left(\mathrm{Rcum}\right)\%=\frac{W_{t}}{W_{o}}\times 100$$

where W_t_ is the cumulative amount of urea released at a time (t) and ${\;W}_{o}$ is the total amount of urea released.

## 3. Results and Discussion

Paying attention to agricultural soil in terms of providing water for irrigation and important nutritional elements for plants like fertilizers is considered one of the important basics in agricultural science. Hydrogels have the capacity to achieve many important and necessary applications, including agricultural uses. The goal of this study was to prepare the following PVA-co-GA/CH and CS-co-GA/PH hydrogels and mix them in low percentages with agricultural soil to examine their capabilities to retain water for long periods of time, in addition to loading them with urea, a common fertilizer, and releasing it to the plants.

## 4. Characterization of the Hydrogels

### 4.1. FTIR Spectroscopy

**Figure 1:**
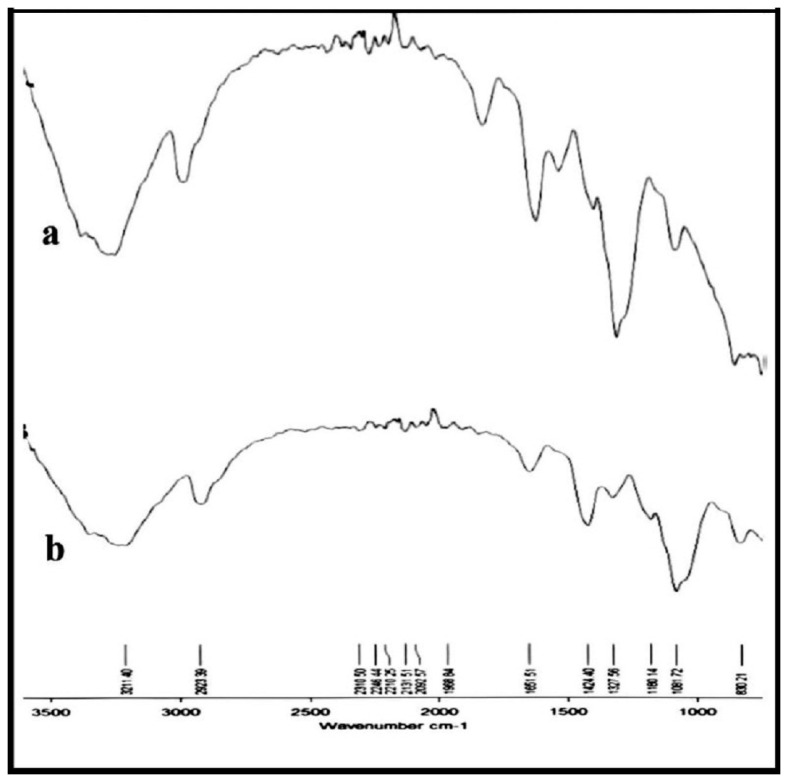
FTIR spectra of (**a**) PVA/GA-CH and (**b**) CS/GA-PH hydrogels.

The FTIR spectrum (Figure 1a,b) shows the absorption frequencies of the main functional groups of the prepared hydrogels. Moreover, the important FTIR characteristic frequencies are recorded in Table 1. The absorption frequency of the PVA/GA-CH hydrogel (Figure 1a and Table 1) at 3374 cm^−1^ is representative of the hydroxyl groups of both PVA and GA.

The band at 1538 cm^−1^ belongs to the amide group of the cross-linker MBA. The absorption frequency at 1649 cm^−1^ belongs to the carbonyl groups of the GA, and the bands at 2941 cm^−1^ and 2844 cm^−1^ represent the methylene groups present in the PVA, GA, and MBA. Finally, the absorption frequency at 1060 cm^−1^ belongs to the (-C-O-C-) of the glucopyranose units of the GA, and those at 1084 cm^−1^ and 1426 cm^−1^ belong to the (-C-O-) and (-C-OH), respectively, of both the PVA and GA.

The CS/GA-PH hydrogel shows, via FTIR, functional groups (Figure 1b, Table 1) similar to the aforementioned functional groups of the PVA/GA-CH hydrogel except those of PVA beside the important functional groups of chitosan, resulting in absorption frequencies at 1633 cm^−1^ and 1531 cm^−1^, which represent the carbonyl groups (amide-I) and the amine group (amide-II), respectively, and absorption frequencies at 1239 cm^−1^, 1061 cm^−1^, and 740 cm^−1^, which belong to the υ(P-O-P) of the ionic cross-linker SHMP.

### 4.2. ^1^H NMR Spectroscopy

**Figure 2:**
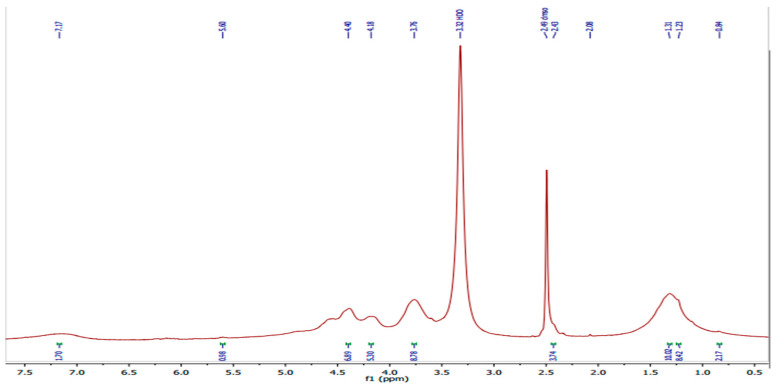
^1^H NMR spectrum of PVA/GA-CH hydrogel.

^1^H NMR of PVA/GA-CH hydrogel (Figure 2 and Table 2) have shown the resonance of (3H, s) at 0.84 ppm which belongs to the methyl protons of the cyclic stereoisomer in a numeric region of the GA sugar. Meanwhile, the resonance of (2H, m) at 1.23 and 1.31 ppm belongs to the methylene protons of PVA, MBA beside those protons present in arabinose sugar of GA. The resonance of (1H, s) at 2.08 ppm belongs to the methine proton of the MBA, while the resonance of (2H, s) at 2.43 ppm and the resonance of (1H, m) at 3.76 ppm are of the methylene and methine protons of the PVA. The resonance of (1H, m) at (4.18 and 4.40) ppm are belongs to hydroxyl proton of PVA and the resonance protons of galactopyranose beside those of arabinose sugars present in GA. Finally, the resonance of (1H, s) at 5.60 ppm represents numeric regions that belong to the cyclic stereoisomer of the GA.

### 4.3. Thermal Analysis Studies

**Figure 3:**
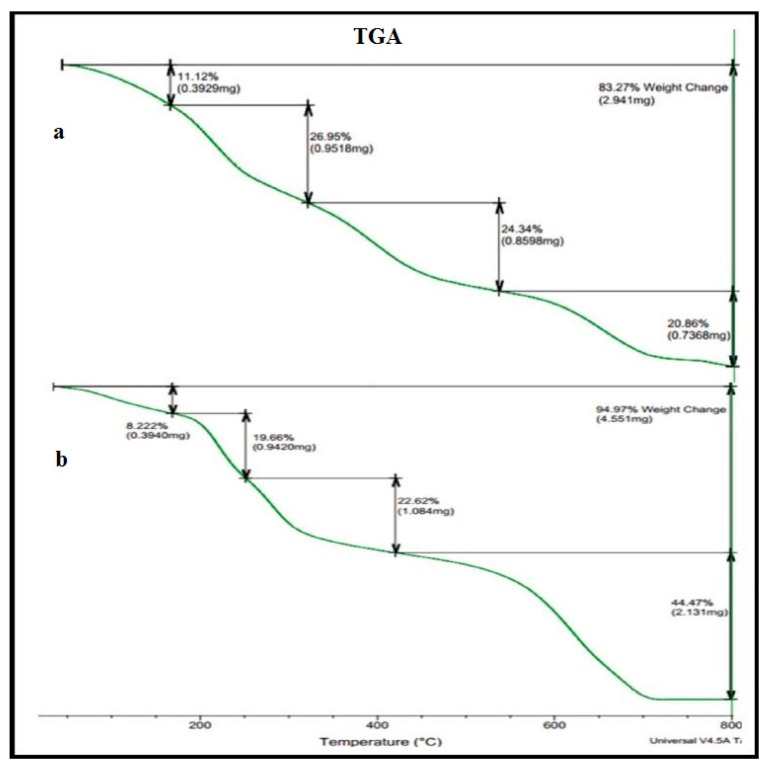
TGA thermograms of (**a**) PVA/GA-CH and (**b**) CS/GA-PH hydrogels.

The TGA and DSC of the PVA/GA-CH and CS/GA-PH hydrogels were studied (Figure 3 and Table 3), and the TGA analysis data for the PVA/GA-CH hydrogel (Figure 3a and Table 3) show that the sample has high thermal stability; the 4.0% weight loss of the hydrogel at 108.4 °C represents the initial decomposition temperature (IDT), and it belongs to the free water in the sample. In contrast, the 82.0% weight loss at 735 °C and the final decomposition temperature (FDT) represent the bonded water (hydrogen bonding). In addition, the 52.5% weight loss at 422 °C represents the maximum decomposition temperature (T_max_) at which the hydrogel degraded, while the crystalline decomposition temperature (T_cr_) at 800 °C has shown 83.3% weight loss where the hydrogel sample was melt.

The thermal analysis of the CS/GA-PH hydrogel (Figure 3b and Table 3) shows its high thermal stability; the TGA (Table 3) shows that the weight loss values occur at lower percentages in comparison with the TGA values of the PVA/GA-CH hydrogel. This is based on the fact that ionic bonds are more stable than covalent bonds. While at almost all decomposition temperatures (IDT, FDT, T_max_ and T_cr_) of CS/GA-PH hydrogel (Table 3) there are depressed in temperatures, and this is because PVA composite structure is more crystalline compering with the structure of chitosan and its architectural symmetry will facilitate the close packing of its chains.

**Figure 4:**
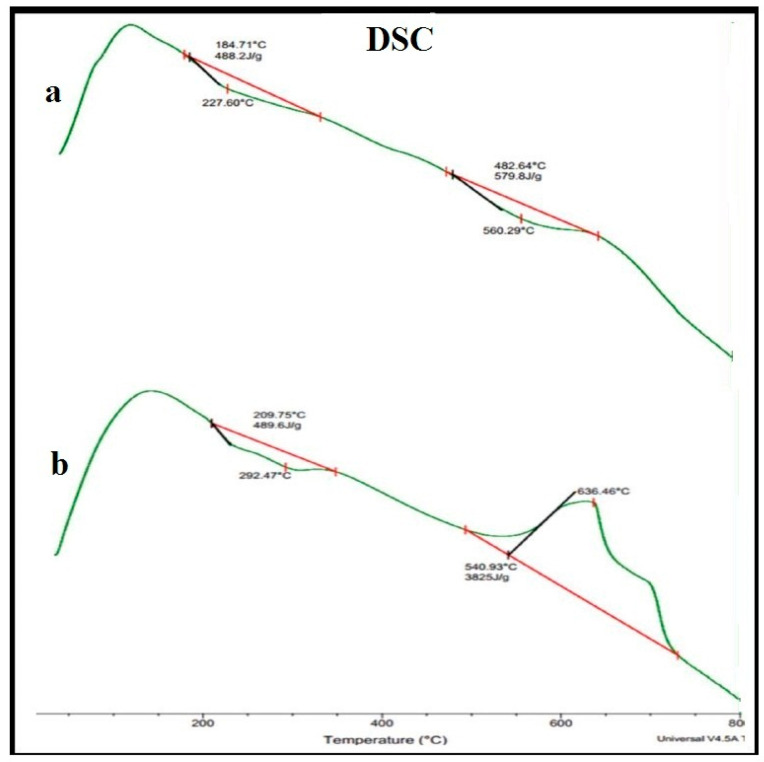
DSC thermograms of (**a**) PVA/GA-CH and (**b**) CS/GA-PH hydrogels.

The DSC thermogram shows the endothermic behavior of the PVA/GA-CH hydrogel (Figure 4a, Table 3), mainly at two curve positions. Where the thermogram shows endothermic heats of fusion (∆H_f_) of +488.2 and +579.8 J/g at 184.7 °C and 482.6 °C, respectively, it indicates that the PVA/GA-CH hydrogel already has a crystalline structure with a stable composition due to the presence of the PVA polymer in its structure. Therefore, it needs +488.2 and +579.8 J/g for decomposition. Even the CS/GA-PH hydrogel shows a DSC thermogram (Figure 4b and Table 3) curve with one endothermic heat of fusion of +489.6 J/g at 209.8 °C and another high value for an exothermic heat of fusion of −3825 J/g at 540.9 °C. In general, the first endothermic ∆H_f_ of the CS/GA-PH hydrogel at 209.8 °C belongs to the decomposition of its ionic structure, while the second curve of the high exothermic heat of fusion value is due to the presence of chitosan in the hydrogel and also because of its non-crystalline structure.

### 4.4. XRD Studies

**Figure 5:**
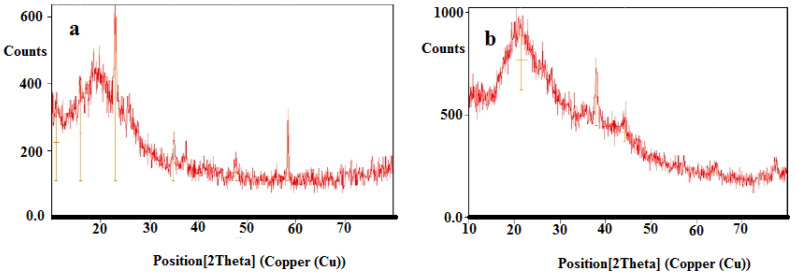
XRD patterns of (**a**) PVA/GA-CH and (**b**) CS/GA-PH hydrogels.

The X-ray diffraction pattern of the PVA/GA-CH hydrogel (Figure 5a) shows that it has a crystalline structure with many positions along the 2θ axes that return to the crystalline structure of the PVA [19]. In general, the maxima at 10.92°, 15.85°, and 22.95° at heights of 230.0, 290.6, and 492.5 counts, respectively, represent the interchain and intrachain polymer interactions of the PVA due to its hydrogen bonding. In addition, the d-spacing of the aforesaid maxima which is representing the distance between planes of atoms is (8.12, 5.59, and 3.88) °A is proportional to the number of electrons or atoms in the structure. The covalent three-dimensional structure of the PVA/GA-CH hydrogel further enhances the crystalline structure of the hydrogel. In contrast, the XRD pattern of the CS/GA-PH hydrogel (Figure 5b) also shows a crystalline structure but with fewer maxima in its XRD diffractogram due to the absence of PVA and the amorphous structures of both the chitosan and gum Arabic. However, only one intense maximum is present at 21.49° at a height of 296.39 counts and with a d-spacing of 4.14 °A, representing the crystalline structure formed by the ionic cross-linker SHMP, which could enhance the crystalline form of the hydrogel structure through its ionic interactions [20].

### 4.5. SEM Studies

**Figure 6:**
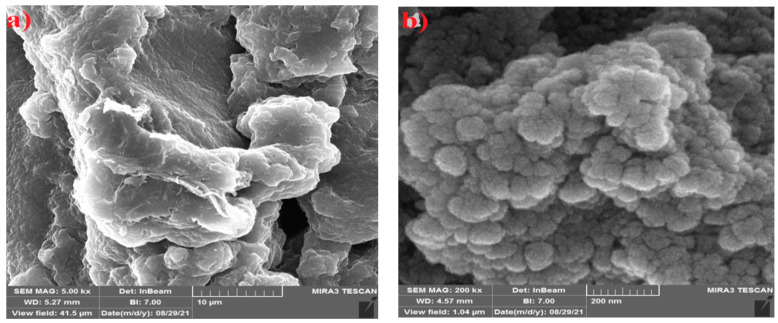
SEM images of (**a**) PVA/GA-CH and (**b**) CS/GA-PH hydrogels.

The SEM images of the PVA/GA-CH and CS/GA-PH hydrogel microspheres were studied, and the SEM image of the PVA/GA-CH hydrogel (Figure 6a) shows a corrugated and folded surface of a homogenous composite with holes, in addition to an irregular morphological surface. The white portion indicates the presence of the crystalline region in the hydrogel specimen.

The SEM image of the CS/GA-PH hydrogel (Figure 6b) shows a gathering of clusters of particles that have microspherical shapes, and the morphological surface shows a low crystalline structure with a homogeneous composition.

### 4.6. Swelling Studies

**Figure 7:**
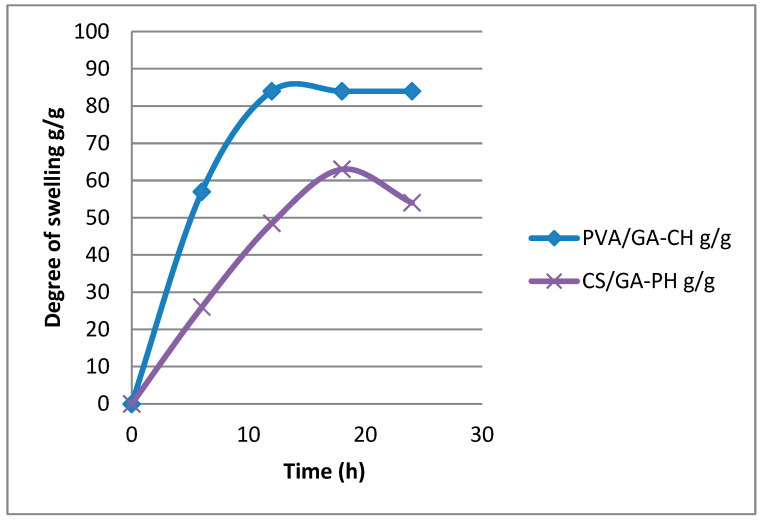
Degree of swelling versus time (h) of the PVA/GA-CH hydrogel microspheres in a RW swelling medium at 10 °C, and of the CS/GA-PH hydrogel microspheres in a swelling medium at a pH 9 and 30 °C.

In general, the prepared hydrogels yielded disparate DS readings in different swelling media and in different ranges of temperatures. However, the PVA/GA-CH hydrogel microspheres showed (Figure 7) a high DS value of 84 g/g in RW at 10 °C, which means that the functional groups of the hydrogel were hydrolyzed and created ionic repulsion forces between the hydrogel chains, finally elevating the microspheres’ degree of swelling [21,22]. In comparison, the DS in the buffered solution media (at pH 4 and pH 9) was lower because the PVA has a basic nature, while gum Arabic has an acidic nature; therefore, the functional groups of the PVA/GA-CH hydrogel in media of both pH values were hydrolyzed and caused ionic attractions between the hydrogel chains, resulting in a depression in the degree of swelling [23].

On the other hand, the maximum DS of the CS/GA-PH hydrogel was 63 g/g in a solution with a pH 9 at 30 °C, whereas it had a lower DS than the maximum DS of the PVA/GA-CH hydrogel. The CS/GA-PH hydrogel was ionically cross-linked through electronic interactions between sodium hexametaphosphate and the polymer’s ions. Repulsion occurred between the SHMP anions and the anions of the gum Arabic; moreover, the attraction occurring between the mentioned anions and the cations of the chitosan functional groups created a degree of swelling in the hydrogel chains. However, this case required a basic swelling media at a pH 9 to neutralize the sodium ions; therefore, the DS of the hydrogel was found to be very low in the swelling medium at a pH 4 and even in the RW medium [24].

### 4.7. Water-Retention Studies

The levels of water retention in agricultural soil specimens mixed with suitable percentages of the hydrogels were studied. In general, agricultural soil has a limited capacity for retaining water, especially in hot regions. Meanwhile, hydrogels can imbibe many times their weight in water and retain the water for a long time without dispensing it readily [25]; therefore, hydrogel microspheres can aid in the irrigation of arid and semi-arid regions.

**Figure 8:**
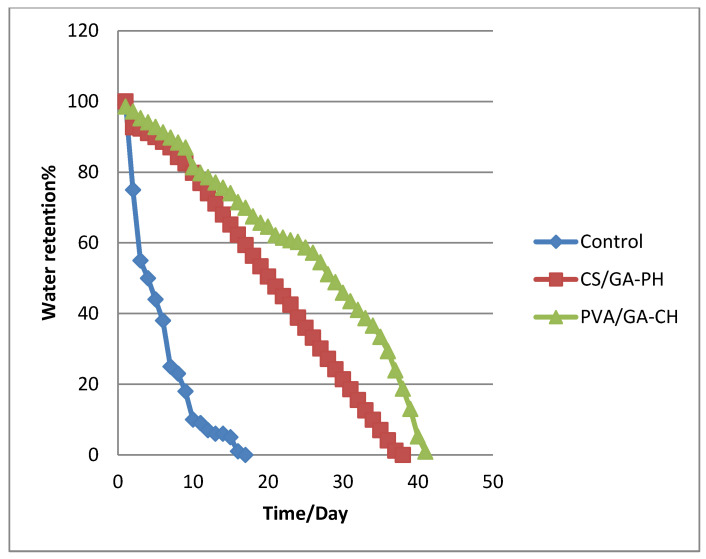
Water-retention (%) of soil/hydrogel composites with time (days) at 20 °C under 25% humidity for the PVA/GA-CH and CS/GA-PH hydrogels.

Therefore, the water retention percentages (WR%) of composites comprising a mixture of agricultural soil with either the PVA/GA-CH hydrogel or the CS/GA-PH hydrogel were studied based on Equation (2). The soil/PVA/GA-CH hydrogel microsphere composite showed the retention of water inside the soil for 42 days; moreover, it maintained the soil’s moisture for the same period (Figure 8). In contrast, the soil/CS/GA-PH hydrogel microsphere composite showed the retention of water inside the soil for 38 days (Figure 8). At the same time, a control sample containing no hydrogel microspheres showed water retention inside the soil sample under the same conditions for only 20 days. The water-retention percentage values of the studied hydrogels are direct embodiments of the maximum degrees of swelling of the hydrogels in which the PVA/GA-CH hydrogel showed a higher DS than the CS/GA-PH hydrogel.

### 4.8. Loading of Urea Fertilizer on Hydrogel Microspheres

Experimental measurements of the maximum loading percentage (L_max_%) of urea loaded onto the PVA/GA-CH hydrogel microspheres yielded 89% after 12 h, meaning that the hydrogel microspheres reached their maximum degree of swelling after 12 h; the microspheres were able to achieve a high loading percentage. The high loading percentage (L_max_%) was due to the irregular morphology of the PVA/GA-CH hydrogel surface and the corrugates and folds present in the surface beside the holes (Figure 6), which could increase the efficiency of loading on the hydrogel microspheres.

In contrast, after 12 h, the L_max_% of urea loaded onto the CS/GA-PH hydrogel microspheres was 79.75%. The lower maximum loading percentage (L_max_%) of the CS/GA-PH hydrogel compared with the L_max_% of the PVA/GA-CH microspheres was also because of their low DS value. However, the cationic nature of chitosan [13] could attract the urea molecules and allow them to reach the cores of the hydrogel microspheres more easily.

### 4.9. Characterization of Loaded Hydrogels

#### FTIR Spectroscopy

**Figure 9:**
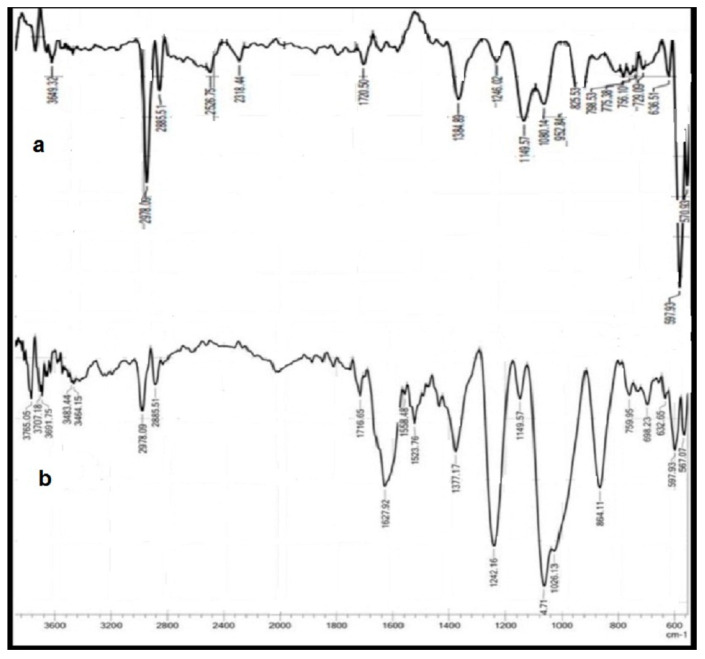
FTIR spectrum of (**a**) PVA/GA-CH/urea and (**b**) CS/GA-PH/urea hydrogels.

The FTIR characteristic frequencies of the important functional groups of the urea-loaded PVA/GA-CH and CS/GA-PH hydrogels are listed in Table 4. The FTIR spectrum of the PVA/GA-CH hydrogel (Figure 9a and Table 4), shows the important functional groups of urea. The absorption bands at (3420 and 1527) cm^−1^ represent the –N-H bonds of the urea. In contrast, the absorption band at 3251 cm^−1^ belongs to the –OH bonds of the PVA and GA polysaccharides. The bands at 1450 and 1080 cm^−1^ belong to the C-N bond of urea. The bands at 1020, 1385, and 826 cm^−1^ belong to the loaded urea.

On the other hand, the FTIR spectrum of the CS/GA-PH hydrogel (Figure 9b, Table 4) shows absorption bands similar to those of the PVA/GA-CH hydrogel (Figure 9a, Table 4); only some extra bands appear at 1242, 727, and 1150 cm^−1^, which represent the -P=O phosphate functional group of the SHMP in the hydrogel.

### 4.10. SEM Studies of Loaded Hydrogels

**Figure 10:**
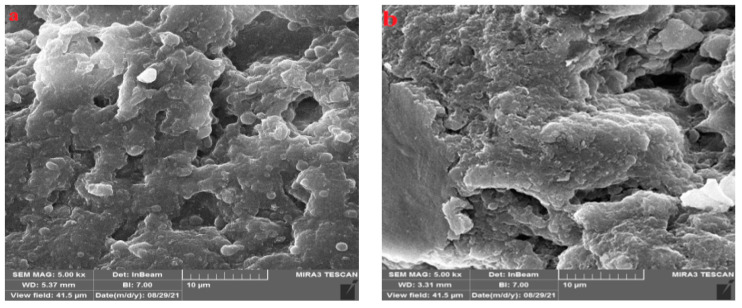
SEM images of loaded hydrogels: (**a**) PVA/GA-CH/urea and (**b**) CS/GA-PH/urea.

The SEM images of the loaded hydrogels show the urea particles interspersed between the folds of the hydrogels. The SEM image of the PVA/GA-CH hydrogel (Figure 10a) after loading shows the crystalline structures of urea, which appear as shining regions nested between the hydrogel folds.

At the same time, the SEM image of the CS/GA-PH hydrogel after loading (Figure 10b) shows the homogeneity of the hydrogel, with urea particles that appear as shiny particles and the composite, which appears as a coherent gel material with many holes.

### 4.11. Release of Urea from the Loaded Hydrogels

**Figure 11:**
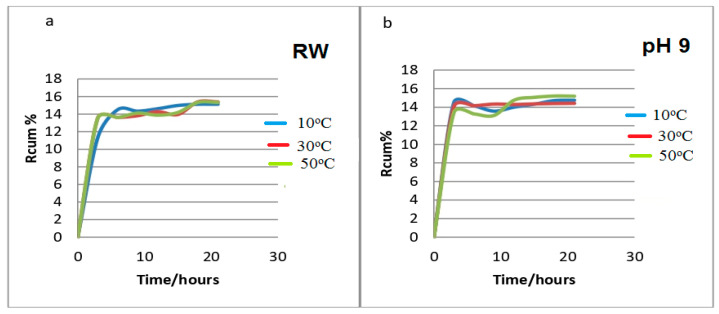
Rcum% of urea from the (**a**) PVA/GA-CH in RW and (**b**) CS/GA-PH in a solution with a pH 9 vs. time (h) at different temperatures.

Loaded urea on PVA/GA-CH and CS/GA-PH hydrogel microspheres has been released and their release behaviors were studied under two variable conditions temperatures and type of solution of release media. Changes in the absorbances of the release solutions with time were measured using a UV-visible spectrophotometer at λ_max_ 278 nm and for a fixed time interval of 3 h. Accordingly, Equation (7) was used to calculate the cumulative release (Rcum%). Experimentally, it was noted that the most suitable release medium for the PVA/CS-CH hydrogel microspheres was the river water (RW) solution at 10 °C (Figure 11a), in which the hydrogel microspheres started to release urea in bursts almost from the beginning of the 5 h of the total release time; then, the release became controlled for almost all of the final 20 h of the total release time. Suitable release conditions occurred in the RW solution at 10 °C; this is because the PVA/CS-CH hydrogel microspheres demonstrated a maximum degree of swelling under such conditions.

In comparison, the CS/GA-PH hydrogel microspheres (Figure 11b) showed maximum release behaviors in a basic release medium with a pH 9 and at a release temperature of 30 °C, where the CS/GA-PH hydrogel microspheres demonstrated their maximum degree of swelling in the pH 9 solution and at 30 °C.

The release curve of the CS/GA-PH hydrogel (Figure 11b) shows more controlled behavior in comparison with the release curve of the PVA/GA-CH hydrogel (Figure 11a). This means that in the pH 9 solution, the CS/GA-PH hydrogel chains necessarily swelled to a point that allowed the polymer to release urea in a controlled manner. Accordingly, the cationic functional groups of chitosan beside the anions of the cross-linker (SHMP) will create balance inside the hydrogel core and finally control the release of the anionic urea [26].

### 4.12. Characterization of the Hydrogels after Release

#### FTIR Spectroscopy

The FTIR spectral data of the PVA/GA-CH and CS/GA-PH hydrogels after release are shown in Table 5. The important characteristic functional groups of the hydrogels are free of urea. Certainly, the presence of the main functional groups of the hydrogel beside its cross-linker after releasing processes, means that the hydrogel is able to retain its basic composition for the subsequent loading and releasing of agrochemicals and for several cycles.

### 4.13. SEM Studies

**Figure 12:**
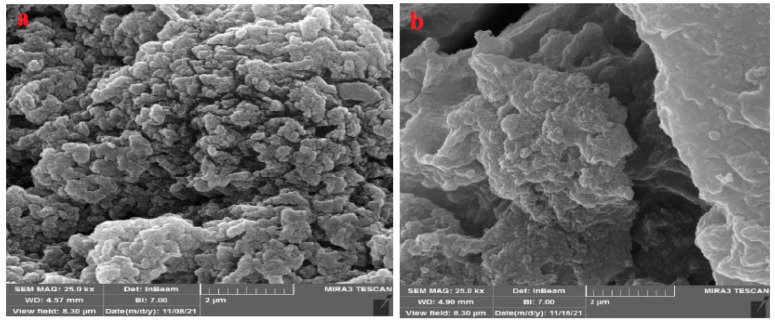
SEM images of (**a**) PVA/GA-CH and (**b**) CS/GA-PH hydrogels after urea release.

The SEM images of the studied hydrogels were taken after release have shown their ability to retain the hydrogel composites complete and well knit. Moreover, the SEM image of the PVA/GA-CH hydrogel (Figure 12a) shows that the microspheres contain empty holes, which most probably represent locations of the released urea molecules. The appearance of the homogeneous surface morphology of the hydrogel in the SEM image in (Figure 12a) provides indications that the loaded urea was released and that the hydrogel microspheres can be reused in drug delivery systems several times.

On the other hand, the SEM image of the CS/GA-PH hydrogel (Figure 12b) shows a more homogeneous morphological surface with deep holes and folds in the hydrogel composite. In addition, the SEM image (Figure 12b) shows a pure hydrogel form with no crystalline particles of urea and its composition is compact and flexible. Accordingly, both hydrogels are suitable for loading and releasing several times.

## 5. Conclusions

The current study investigated the exploitation of the large-scale water-storage tendency of hydrogels, in addition to their capability to load agrochemicals at high percentages and retain them for release in a controlled manner over a long period. In general, two hydrogel systems were prepared from gum Arabic which was blended first with PVA and second with chitosan. The PVA/GA hydrogel was prepared, and the covalent cross-linker glutaraldehyde was used. Meanwhile, the CS/GA hydrogel was prepared, and an ionic cross-linker was used.

Both the PVA/GA-CH and CS/GA-PH hydrogels were characterized, and their structures were examined. FTIR spectroscopy showed that the hydrogels’ functional groups appeared at their characteristic absorption frequencies. ^1^H NMR spectroscopy showed the resonances of the hydrogel protons appearing at their characteristic chemical shifts. The thermal TGA and DSC analyses showed that both hydrogels are thermally stable. The TGA analysis of the PVA/GA-CH hydrogel showed higher decomposition temperatures due to the symmetry of the PVA structure, while the CS/GA-PH hydrogel demonstrated less weight loss (%) because of its ionic bonds, which are more stable than covalent bonds. The DSC analysis showed that the PVA/GA-CH hydrogel demonstrates endothermic heat fusion, whereas the CS/GA-PH hydrogel has one exothermic heat fusion curve that is due to the decomposition of the non-crystalline structure of the chitosan. The XRD pattern of PVA/GA-CH hydrogel it shows crystalline structure return to the PVA symmetry structure and the three dimensional structure done by the covalent cross-linker, while CS/GA-PH hydrogel has less crystalline maxima done by its ionic crosslinking. The SEM images of both hydrogels showed homogeneous composites with holes and folds in their surface morphologies.

The maximum degree of swelling of the hydrogel microspheres showed a great variation depending on the composite, the electronic interactions between their chains, and the swelling media solution and its temperature. Therefore, the PVA/GA-CH hydrogel yielded a DS = 84 g/g in RW at 10 °C, while the CS/GA-PH hydrogel had a DS = 63 g/g at a pH 9 at 30 °C. The electronic interactions between the hydrogel composite, in addition to its cross-linker and the ions of the swelling media, fixed the suitable conditions for each hydrogel to reach its maximum DS.

Both hydrogels showed high levels of water retention in agricultural soil: the soil/PVA/GA-CH composite demonstrated water retention for 42 days, whereas the soil/CS/GA-PH composite retained water for 38 days; this proved that the degree of swelling of the hydrogels plays a significant role in water retention inside the soil. The L_max_% of the PVA/GA-CH hydrogel microspheres with urea was 89%, and this high percentage was due to the irregular morphology of the hydrogel surface and the presence of corrugates and folds in its surface, which could increase its efficiency of loading. In comparison, the L_max_% of the CS/GA-PH hydrogel with urea was 79.75%; this is still high because of the cationic nature of chitosan, which could attract the urea molecules and allow them to reach the cores of the hydrogel microspheres more easily. It was shown that the most suitable release medium for the PVA/CS-CH hydrogel microspheres was the river water (RW) solution, and the loaded urea was released in bursts for 5 h and then under controlled release for 20 h at 10 °C; this was according to the swelling conditions. In comparison, the CS/GA-PH hydrogel microspheres showed that their best release medium had a pH 9 and a temperature of 30 °C, and this was also according to the swelling conditions. Finally, the release curve of the CS/GA-PH hydrogel demonstrated more controlled behavior in comparison with the release curve of the PVA/GA-CH hydrogel because in a solution at a pH 9, the ionic hydrogel chains could control the electronic interactions and swelling to a limit that allowed the polymer to control its release more. Accordingly, the cationic functional groups of chitosan beside the anions of the cross-linker (SHMP) create a balance inside the hydrogel core and ultimately control the release of the anionic urea. All the characteristic analyses of the hydrogel microspheres show that they can be reused in drug delivery systems several times.

## Figures and Tables

**Table 1:** FTIR characteristic frequencies of the main functional groups of the prepared hydrogels.

FTIR Main Functional Groups										
Sample	υ(C-H)_str_	Amide-I	Amide-II	υ(C=O)_str_ _Asym. α sym._	υ(O-H)_str_	υ(C-OH)_def_	υ(C-O)_str_	υ(C-O-C)	υ(N-H)_str_	υ(P-O-P)
Wave number/cm^−1^										
PVA/CS-CH	2844 2941	---------	--------	1649	3374	1426	1084	1060	3361	--------
CS/GA-PH	2876 2941	1636	1524	---------	3364	1380	1084	1003	3364	1239, 1061, 740

**Table 2:** ^1^H NMR results for PVA/GA-CH hydrogel.

Sample	Chemical Shift σ/ppm	Description of Proton
PVA/GA-CH	0.84	Methyl protons; cyclic stereoisomer
	1.23 and 1.31	Methylene protons; arabinose sugar
	2.08	Proton next to C=O and NH
	2.43	Methylene protons
	3.76	Methine protons; rhamnose sugar
	4.18 and 4.40	Alcoholic protons present in galactopyranose and arabinose sugars
	5.60	A cyclic stereoisomer of gum arabic

**Table 3:** TGA and DSC thermal analysis data of the prepared hydrogels.

Sample	TGA Weight Loss (%)				DSC (W/g)		
	IDT °C	FDT °C	T_max_ °C	T_cr_ °C	Tg °C	∆H_f_ (J/g)	
PVA/GA-CH	4.0%	82.0%	52.5%	83.3%	108.4/°C	+488.2	+579.8
	108.4/°C	735.0/°C	422.0/°C	800.0/°C		184.7/°C	482.6/°C
CS/GA-PH	2.5%	94.0%	50.0%	95.0%	96.3/°C	+489.6	−3825.0
	96.3/°C	715.0/°C	405.0/°C	608.0/°C		209.8/°C	540.9/°C

**Table 4:** FTIR characteristic frequencies of the important functional groups of the hydrogels loaded with urea.

FTIR Main Functional Groups								
Sample	υ(C-H)_str_	υ(C=O)_str_ _Asym. α sym._	υ(O-H)_str_	υ(C-N)_def_	υ(N-O)_str_	υ(C-O-C)	υ(N-H)_str_	υ(P-O-P)
Wave number/cm^−1^								
PVA/CS-CH	2886, 2978	1675	3251	1450, 1080	1020, 1385, 826	1060	1527, 3420	--------
CS/GA-PH	2886 2978	1628	3210	1436, 1085	1026, 1377, 864	1003	1524 3464	1242, 1152, 727

**Table 5:** FTIR characteristic frequencies of the important functional groups of the used hydrogels after release.

Sample	FTIR Functional Group							
	υ(C-H)_str_	υ(C=O)_str_	υ(O-H)_str_	υ(C-OH)_def_	υ(C-O)_str_	υ(C-O-C)_str_	υ(N-H)_str_	υ(P-O-P)
Wave number/cm^−1^								
PVA/CS-CH	2860, 3031	1610	3224	1348	1085	1030	1520	--------
CS/GA-PH	2862 2931	1645	3244	1380	1079	1020	1552	1020, 1149, 1255

## References

[1] Kabir M.H., Ahmed K., Furukawa H. (2017). A low cost sensor based agriculture monitoring system using polymeric hydrogel. J. Electrochem. Soc..

[2] Ajuik T.A., Nokes S.E., Montross M.D., Wendroth O. (2022). The Impacts of Bio-Based and Synthetic Hydrogels on Soil Hydraulic Properties. Polymer.

[3] Neethu T.M., Dubey P.K., Kaswala A.R. (2018). Prospects and applications of hydrogel technology in agriculture. Int. J. Curr. Microbiol. Appl. Sci..

[4] Jeong D., Kim C., Kim Y., Jung S. (2020). Dual crosslinked carboxymethyl cellulose/polyacrylamide interpenetrating hydrogels with highly enhanced mechanical strength and superabsorbent properties. Eur. Polym. J..

[5] Garbowski T., Bar-Mickalczyk D., Charazirnska S., Grabowska-Polanowska B., Kowalczyk A., Lochynski P. (2023). An Overview of Natural Soil Amendments in agriculture. Soil Tillage Res..

[6] Saha A., Sekharan S., Manna U. (2020). Superabsorbent Hydrogel (SAH) as a SOIL Amendment for Drought Management: A Review. Soil Tillage Res..

[7] Hasan H.H., Razali M., Fatin S., Muhammad N.S., Ahmad A. (2019). Research Trends of Hydrological Drought: A Systamatic Review. Water.

[8] Smagin A., Sadovnikova N., Smagina M. (2019). Synthetic Gel Structures in Soil for Sustainable Potato Farming. Sci. Rep..

[9] Palmqvist N.G.M. (2017). Nanoparticles: Case Studies of their Synthesis, Properties and Biological Interaction. Ph.D. Thesis.

[10] Park S., Park K.M. (2016). Engineered polymeric hydrogels for 3D tissue models. Polymers.

[11] Fan J., Li G., Deng S., Wang Z. (2019). Mechanical Properties and Microstructure of Polyvinyl alcohol (PVA) Modified Cement Mortar. Appl. Sci..

[12] Brzyski P. (2021). The influence of Gum Arabic Admixture on the Mechanical Properties of Lime-Metakaolin Paste Used as Binder in Hemp Concrete. Materials.

[13] Chen Y., Zhang Q., Zhang Y., Wei P., Yu X., Huang J., Cai J. (2021). Super-Strong and Super-Stiff Chitosan Filaments with Highly Ordered Hierarchical Structure. Adv. Funct. Mater..

[14] Rynkowska E., Fatyeyeva K., Marais S., Kujawa J., Kujawski W. (2019). Chemically and Thermally Crosslinked PVA-Based Membranes: Effect on Swelling and Transport Behavior. Polymer.

[15] Wu L., Liu M. (2008). Preparation and Characterization of Cellulose Acetate-coated Compound Fertilizer with Controlled–release and Water Retention. Polym. Adv. Technol..

[16] Mutlaq M.S., Jabrail F.H. (2022). Controlled Delivery System for NPK Agrochemical Release from Chitosan Copolymer Hydrogels. Am. J. Appl. Sci..

[17] Gupta K.C., Jabrail F.H. (2007). Glutaraldehyde Cross-Linked Chitosan Microspheres for Controlled Release of Centochroman. Carbohydr. Res..

[18] Gupta K.C., Jabrail F.H. (2007). Controlled-Release Formulations for Hydroxy Urea and Rifampicin Using Polyphosphate-Anion-Crosslinked Chitosan Microspheres. J. Appl. Polym. Sci..

[19] Gaaz T.S., Sulong A.B., Akhtar M.N., Kadhum A.H., Mohamad A.B., Al-Amiery A. (2015). Properties and Applications of Poly (vinyl alcohol) Halloysite Nanotubes and their Nanocomposites. Molecules.

[20] Jampafuang Y., Tongta A., Waiprib Y. (2019). Impact of crystalline structural differences between α-and β-chitosan on their nanoparticle formation via ionic gelation and superoxide radical scavenging activities. Polymers.

[21] Djumaev A., Tashmukhamedova S. (2020). Physical and chemical properties of PVA-CMC based hydrogel carrier loaded with herbal hemostatic agent for application as wound dressings. Natl. J. Physiol. Pharm. Pharmacol..

[22] Mazuki N.F., Majeed A.A., Nagao Y., Samsudin A.S. (2020). Studies on ionics conduction properties of modification CMC-PVA based polymer blend electrolytes via impedance approach. Polym. Test..

[23] Žuržul N., Ilseng A., Prot V.E., Sveinsson H.M., Skallerud B.H., Stokke B.T. (2020). Donnan Contribution and Specific Ion Effects in Swelling of Cationic Hydrogels are Additive: Combined High-Resolution Experiments and Finite Element Modeling. Gels.

[24] Jastram A., Lindner T., Luebbert C., Sadowski G., Kragl U. (2021). Swelling and Diffusion in Polymerized Ionic Liquids-Based Hydrogels. Polymers.

[25] Kabiri K., Zohuriaan-Mehr M.J. (2003). Superabsorbent hydrogel composites. Polym. Adv. Technol..

[26] Hu K., Kong M., Qin M., Zeng J., Ai B., Zhang J., Zhang H., Zhong F., Wang G., Zhuang L. (2021). Experimental and Theoretical Studies of Chitosan Dissolution in Ionic Liquids: Contribution Ratio Effect of Cations and Anions. J. Mol. Liq..

